# Lipid-Iron Nanoparticle with a Cell Stress Release Mechanism Combined with a Local Alternating Magnetic Field Enables Site-Activated Drug Release

**DOI:** 10.3390/cancers12123767

**Published:** 2020-12-14

**Authors:** Tuula Peñate Medina, Mirko Gerle, Jana Humbert, Hanwen Chu, Anna-Lena Köpnick, Reinhard Barkmann, Vasil M. Garamus, Beatriz Sanz, Nicolai Purcz, Olga Will, Lia Appold, Timo Damm, Juho Suojanen, Philipp Arnold, Ralph Lucius, Regina Willumeit-Römer, Yahya Açil, Joerg Wiltfang, Gerardo F. Goya, Claus C. Glüer, Oula Peñate Medina

**Affiliations:** 1 Section Biomedical Imaging, Department of Radiology and Neuroradiology Universitätsklinikum Schleswig-Holstein Campus Kiel, Christian Albrechts Universität zu Kiel, 24105 Kiel, Germany; tuula.penate@rad.uni-kiel.de (T.P.M.); jana.humbert@rad.uni-kiel.de (J.H.); akoepnick@pharmazie.uni-kiel.de (A.-L.K.); reinhard.barkmann@rad.uni-kiel.de (R.B.); olga.will@rad.uni-kiel.de (O.W.); timo.damm@rad.uni-kiel.de (T.D.); glueer@rad.uni-kiel.de (C.C.G.); 2 Institute for Experimental Cancer Research, Christian-Albrechts-University Kiel, 24105 Kiel, Germany; lia.appold@rwth-aachen.de; 3 Klinik für Mund-, Kiefer- und Gesichtschirurgie, Universitätsklinikum Schleswig-Holstein Campus Kiel, Christian Albrechts Universität zu Kiel, 24105 Kiel, Germany; mirko.gerle@mkg.uni-kiel.de (M.G.); chuhanwen@zju.edu.cn (H.C.); nmpurcz@gmx.de (N.P.); Yahya.acil@mkg.uni-kiel.de (Y.A.); wiltfang@mkg.uni-kiel.de (J.W.); 4 Department of Oral and Maxillofacial Surgery, Second Affiliated Hospital, Zhejiang University, Hangzhou 310058, China; 5 Helmholtz-Zentrum Geesthacht, Zentrum für Material- und Küstenforschung GmbH, Max Planck Straße 1, 21502 Geesthacht, Germany; vasyl.haramus@hzg.de (V.M.G.); regine.willumeit@hzg.de (R.W.-R.); 6 Institute of Nanoscience of Aragon (INA) and Condensed Matter Physics Dept., University of Zaragoza, C.P. 50.018 Zaragoza, Spain; beasanz@unizar.es (B.S.); goya@unizar.es (G.F.G.); 7 Cleft Palate and Craniofacial Center, Department of Plastic Surgery, Helsinki University Hospital, 00029 HUS Helsinki, Finland; juho.o.suojanen@hus.fi; 8 Päijät-Häme Joint Authority for Health and Wellbeing, Department of Oral and Maxillo-Facial Surgery, 15850 Lahti, Finland; 9 Anatomical Institute, Christian-Albrechts-University Kiel, 24105 Kiel, Germany or Philipp.Arnold@FAU.de (P.A.); rlucius@anat.uni-kiel.de (R.L.)

**Keywords:** theranostic, stimuli responsive release, liposome, acid sphingomyelinase (ASMase), drug delivery, molecular imaging, magnetic field

## Abstract

**Simple Summary:**

A novel active release system magnetic sphingomyelin-containing liposome encapsulated with indocyanine green, fluorescent marker, or the anticancer drug cisplatin was evaluated. The liposomal sphingomyelin is a target for the sphingomyelinase enzyme, which is released by stressed cells. Thus, sphingomyelin containing liposomes behave as a sensitizer for biological stress situations. In addition, the liposomes were engineered by adding paramagnetic beads to act as a receiver of outside given magnetic energy. The enzymatic activity towards liposomes and destruction caused by the applied magnetic field caused the release of the content from the liposomes. By using these novel liposomes, we could improve the drug release feature of liposomes. The improved targeting and drug-release were shown in vitro and the orthotopic tongue cancer model in mice optical imaging. The increased delivery of cisplatin prolonged the survival of the targeted delivery group versus free cisplatin.

**Abstract:**

Most available cancer chemotherapies are based on systemically administered small organic molecules, and only a tiny fraction of the drug reaches the disease site. The approach causes significant side effects and limits the outcome of the therapy. Targeted drug delivery provides an alternative to improve the situation. However, due to the poor release characteristics of the delivery systems, limitations remain. This report presents a new approach to address the challenges using two fundamentally different mechanisms to trigger the release from the liposomal carrier. We use an endogenous disease marker, an enzyme, combined with an externally applied magnetic field, to open the delivery system at the correct time only in the disease site. This site-activated release system is a novel two-switch nanomachine that can be regulated by a cell stress-induced enzyme at the cellular level and be remotely controlled using an applied magnetic field. We tested the concept using sphingomyelin-containing liposomes encapsulated with indocyanine green, fluorescent marker, or the anticancer drug cisplatin. We engineered the liposomes by adding paramagnetic beads to act as a receiver of outside magnetic energy. The developed multifunctional liposomes were characterized in vitro in leakage studies and cell internalization studies. The release system was further studied in vivo in imaging and therapy trials using a squamous cell carcinoma tumor in the mouse as a disease model. In vitro studies showed an increased release of loaded material when stress-related enzyme and magnetic field was applied to the carrier liposomes. The theranostic liposomes were found in tumors, and the improved therapeutic effect was shown in the survival studies.

## 1. Introduction

Drug delivery allows a higher local concentration of the drug at the disease site while the side effects are reduced. The delivery implies an improved efficacy and tolerance of existing and demonstrated drugs. By preferentially enhancing the localization of pharmaceutical activity in the organ or tissue of interest, their use can reduce the required systemic doses. Additionally, targeted delivery enables new drugs with nonsuitable pharmacokinetics as a conventional therapy to be implemented for clinical use. A substantial step forward would be a targeted and controlled release system that could increase biologically active molecules at the disease site. The need for targeted and controlled release systems in medicine is not limited to oncology: autoimmune diseases, such as rheumatoid arthritis or sarcoidosis and inflammation diseases, could benefit from the targeted release as well. Nanoparticle- and nanostructure-based delivery systems are widely developed and used to promote the efficacy of drug therapies. Most nanoparticle delivery systems accumulate close to tumors because of the enhanced permeation and retention (EPR) effect [1]. However, some clinical trials have failed because of the poor release of the drugs from the carrier even though the carrier had reached the vicinity of the cancer cells like is the case in liposomal cisplatin [2]. A delivery system that could reliably open on the target site to elevate the biologically available drug would solve this problem. There have been successes in delivering drugs specifically to cells on the site of interest [3,4,5,6,7], but in tumor targeting, there are still problems with tumor pressure, accessibility due to poor vascularization, and blood flow. The tumor entry for delivery vehicles is still partly governed by enhanced permeation and retention that varies between location and tumor type. Thus, new strategies are still needed for targeting and especially for release.

This report proposes a novel delivery and release concept: we equipped a well-established drug carrier system, liposomes, with two independent functions that provide site activated release. One release mechanism reacts to the biological conditions at the disease pathology, and another, electromagnetic mechanism, can be applied in a controlled manner from outside of the body. Liposomes per se cannot substantially penetrate a tumor. We incorporated sphingomyelin (SM) lipid in the membrane of the liposome carriers. When exposed to the stress-related enzyme sphingomyelinase (SMase), sphingomyelin in the liposomal membrane is converted to ceramide. Here we tested the assumption that lipid composition changes affect the membrane characteristics and allow active release from the liposome into the stressed and immunologically active site. 

Acid sphingomyelinase (ASMase) is an attractive new target protein for therapeutic intervention. This protein is tightly controlled in the cytoplasm; however, in various disorders, it is secreted across the cell membrane [8,9]. The ASMase related pathway is one of the routes for cell stress responses [10]: ASMase is spontaneously generated by stressed cells in tumors and inflammation by processes that involve tumor necrosis facto-alfa (TNF-α) and tumor necrosis factor like protein activation [11,12]. Diverse stress stimuli rapidly activate ASMase and promote increased cellular ceramide levels over minutes to hours [10]. The enzyme is activated by various stress inducers, such as oxidative stress [13], ionizing radiation [14,15], and chemotherapeutic agents, such as cisplatin [16], doxorubicin [17], and gemcitabine [18]. The fact that ASMase simultaneously reflects the severity of the disease and can facilitate the delivery system’s activation is a potential missing piece to overcome the previously presented delivery systems’ limitations to deliver drugs and DNA to the right place with sufficient biologically active quantities. It has previously shown that ASMase secretion leads to the conversion of sphingomyelin to ceramide. When enough ceramide is produced, ceramide-rich domains are formed in the model membrane [19] and the cellular context [20]. The formed domains cluster receptor molecules on the cellular membrane, thereby initiating the apoptosis signaling cascade [11,16]. In giant unilamellar vesicle model membranes, ceramide forms solid microdomains [19,21]. The domain formation creates liquid–solid borders, and this phase separation weakens the lipid bilayer [22]. 

We wanted to use the enzymatic activity to weaken the liposomes to release their content in the site where the stress-related enzyme is available. We equipped our liposomes with sphingomyelin. On the other hand, we wanted to bring energy to the system via an alternating magnetic field. Therefore, small 5 nm-sized ferric particles were included in the liposomal membrane to act as energy receivers. Using liposomes as established carriers for cisplatin and indocyanine green (ICG), we added an endogenous switch triggered by an elevated systemic enzyme concentration. An external control-switch, ferric nanoparticles, accelerates the release action when an alternating magnetic field is applied. The liposomes were characterized by physicochemical methods, and the ASMase effects with and without magnetic force on the liposomes were tested in both test tubes and cells. The squamous cell carcinoma (SCC) orthotopic mouth tumor model in mice was used to test theranostic delivery capability in vivo. 

## 2. Results

### 2.1. Biophysical In Vitro Characterization

**Figure 1:**
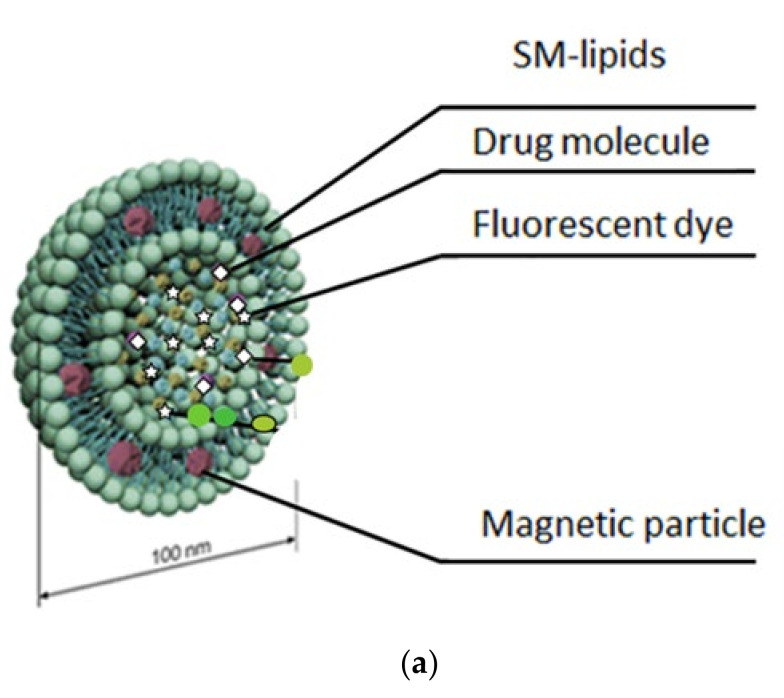

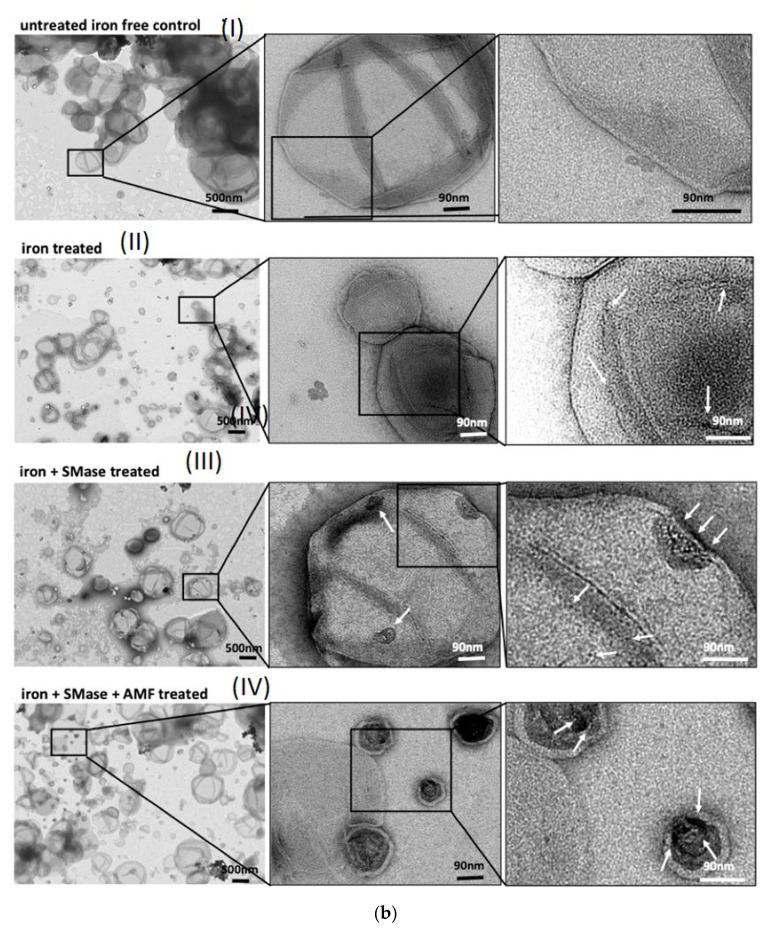

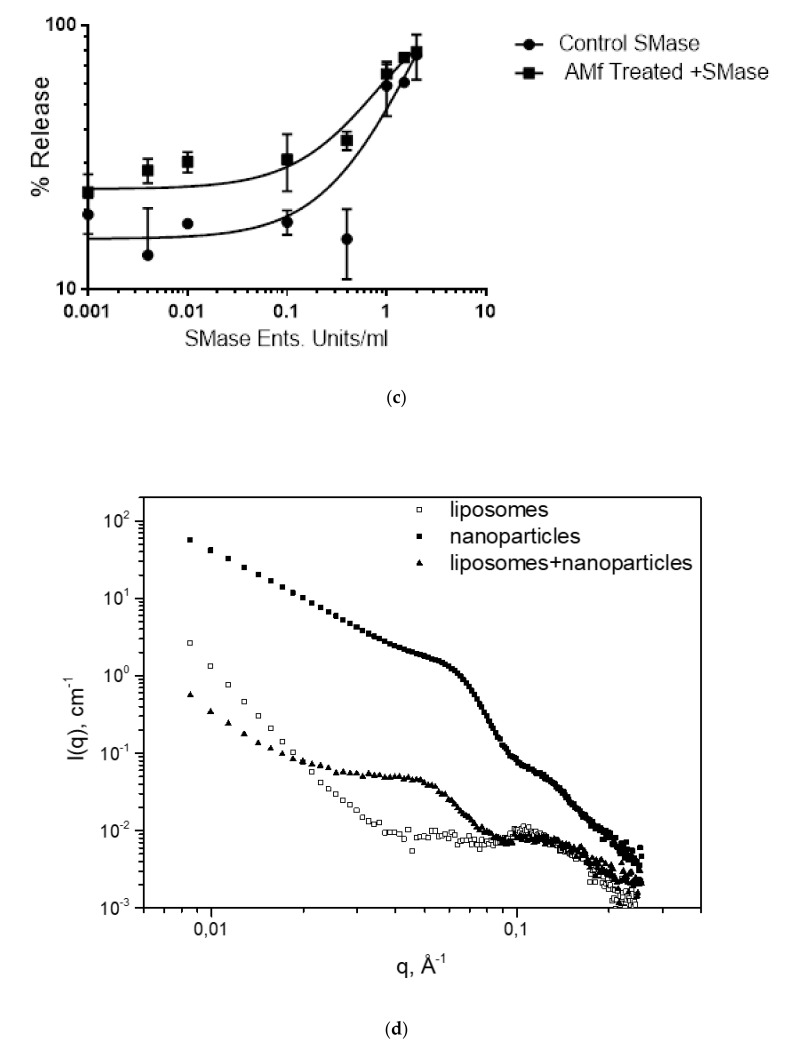

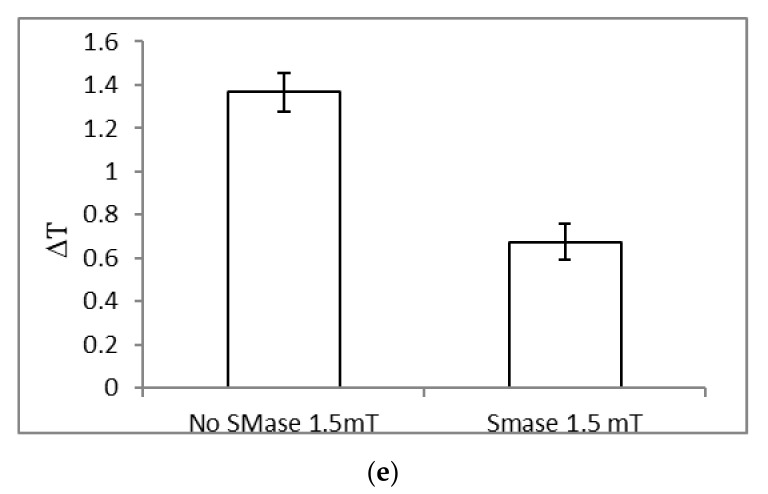
Magnetoenzymatic Sphingomyelin (SM) liposomes. (**a**) Schematic illustration of SM-liposomes containing Fe_3_O_4_-nanoparticles (dark gray) embedded in the lipid membrane and a payload of imaging and/or drug molecule (star and diamond) inside the liposome. (**b**) TEM microscopy of SM liposomes. Liposomes were untreated and iron-free (panel I), untreated and loaded with iron (panel II), or iron-containing and additionally exposed to Sphingomyelinase (SMase) (0.4 U/mL) alone (panel III) or in combination with an AMF (6 mT, 20 min) (panel IV). (**c**) Release of cisplatin from SM-liposomes when the increased dose of SMase was used and AMF treatment was kept constant (20 min, 2 mT, 100 kHz). The (■) shows results for SM-liposomes loaded with cisplatin, containing ferric nanoparticles (DSPC:SM:DOTAP:chol (20:30:20:30) mol/mol), and treated with different concentrations of SMase from 0 to 2 U/mL and AMF. The (●) group shows results for SM-liposomes (DSPC:SM:DOTAP:chol (20:30:20:30) mol/mol) with the same 0–2 U/mL SMase pretreatment but without AMF. After the treatment, liposomal solution was collected and dialyzed overnight against PBS, and the leakage was calculated by measuring the cisplatin absorbance inside and outside of the dialysis bag. Each bar represents the mean ± SEM. Significance determined by *t*-test and indicated by *. (**d**) SAXS spectra of SM liposomes (DSPC:SM:DOTAP:chol, molar % 20:30:20:30, respectively) (□), solution of Fe_3_O_4_ nanoparticles (■) (0.3 mg/mL), and SM liposomes with Fe_3_O_4_ nanoparticles (▲) (0.3 mg/mL) in PBS. The solution temperature is 25 °C. (**e**) Temperature increase (∆T) of the liposome containing buffer after AMF 10-min treatment at RT. With the ASMase treated magneto enzymatic SM liposomes compositions, a 0.70 °C (+/- 0.2 standard deviations) increase in temperature from 24.0 to 24.70 °C was observed from before alternating magnetic field (AMF) stimulation and after commencing 20 min of AMF treatment (t = 20 min). For the non-ASMase-treated MESL compositions, a 1.30 °C (+/- 0.15 standard deviations) increase in temperature from 24.0 to 25.30 °C was observed from before the AMF stimulation and after commencing 20 min of AMF treatment (t = 20 min).

This study’s purpose was to improve the drug release feature of liposomes by developing a liposomal nanoparticle system (for structure, refer to Figure 1a), which can be weakened at the disease site, thereby enabling the release of the contents in a remote-controlled manner. The cisplatin as well as the fluorescent model for drug the ICG [23] was loaded to these magnetoenzymatic (MESL) liposomes. The size of the liposomes was around 110 nm in both formulations. In the giant unilamellar vesicle (GUV) model, we have earlier shown that SM containing liposomes form ceramide rich microdomains and induce budding when treated with sphingomyelinase [19]. In the present study, we demonstrate that the behavior of in situ produced ceramide in the liposome membrane can be utilized in drug-delivery in biologically relevant conditions. The release was improved by bringing energy to the lipid film. Small iron nanoparticles were included in the liposomal formulation to apply an alternating magnetic field to the system (Figure 1a). Small 5 nm core-shell iron nanoparticles were used; previous studies indicate that particles of this size can be incorporated into the lipid membrane [24], which was also demonstrated in our liposomes by transmission electron microscopy (TEM) and small-angle X-ray scattering (SAXS) analysis (Figure 1b,c). We aimed to employ a low but sufficiently high magnetic field that is simultaneously regarded as safe. Thus, 1–6 mT magnetic fields were used for the release studies. These field strengths are considered to be safe and roughly equal to the strengths found around cellular phones. When our liposomes were analyzed with TEM, in addition to the clear association of the 5 nm iron nanoparticles on the membrane, a formation of large agglomerates in the liposomal membrane could be observed after sphingomyelinase treatment (Figure 1b, panel III). In the overview of TEM images, multiple liposomes can be appreciated for each condition. TEM of the different liposome treatments (untreated iron-free panel I, liposomes with iron only panel II, iron-containing liposomes treated with SMase panel III, and iron-containing liposomes treated with SMase and alternating magnetic field (AMF) in panel IV) indicates a similar distribution of liposomes on the TEM grid. At a higher magnification, no particles of high density can be detected in untreated iron-free conditions, and only a very evenly gray background can be observed (Figure 1b, panel I). In the magnified TEM images of the untreated iron-free control liposomes, no iron particles can be detected. In the iron-containing liposomes, small dots appear on the liposomes (white arrows in Figure 1b, panel II), which most likely represent the individual 5 nm iron particles or small clusters. However, they do not form large iron nanoparticle clusters. In the iron-containing SMase-treated sample, individual iron particles and large domain-like clusters of iron particles can be observed (white arrows in Figure 1b, panel III). Following the treatment of iron liposomes with SMase and AMF, an increasing number of small vesicles (≈50 nm) appear, which are darkly stained (Figure 1b, panel IV). In the highest magnification, individual iron particles can be discriminated within these smaller liposomes that appear to be filled with iron (white arrows Figure 1b, panel IV, the image on the right). 

The optimal size for the maximum heating efficiency of an applied magnetic field in Fe_3_O_4_ nanoparticles in an aqueous medium is approximately 18–25 nm [25]. However, it has also been reported that magnetic interactions among nanoparticles produced by clustering can shift this optimum to smaller or larger values, depending on the original single particle size [26]. The average size of iron nanoparticles (d = 5 nm) in our samples was too small for optimal heating performance under AMF, as expected from accepted models on Néel and Brown relaxation contributions [27]. However, the resulting clustering observed within the liposomal membrane resulted in an improved power release (Figure S1). Agglomeration of magnetite nanoparticles (NPs) is an established process that can originate from different mechanisms, including opsonization (in protein-rich media) and electrostatic and dipolar magnetic interactions. In a lipid membrane, there is only space for a limited amount of agglomerated particles, which might constitute an upper limit for the specific power absorption (SPA) values of the liposomal vectors and iron particles (Figure S1). Electrical interactions are challenging to neutralize in solution; therefore, there is always a small, unavoidable degree of agglomeration during the preparation of magnetoliposomes. Although given the small sizes (5 nm) of our particles, no magnetic interaction is expected due to the superparamagnetic fluctuation of the magnetic moments (through both Néel and Brown relaxation processes), magnetic nanoparticles can form self-organized microstructures under the influence of an externally applied magnetic field. Moreover, other researchers and we have previously shown that giant liposomes face a formation of lipid domains when SM containing liposomes are treated with ASMase [19] or when ceramide is added to the membrane [21]. In the TEM images of the ASMase treated liposomes (Figure 1b, Panel III and IV), we observe larger agglomerated rafts or domains on the liposome surface that are more lateral than globular, and patches of iron particles appear on liposomes in addition to single iron particles: in the largest magnification, individual iron particles and clusters of multiple iron particles can be detected (white arrows in Figure 1b, panel III). There is also an inverted curvature present in the iron particle rich domain. In the iron-containing SMase and AMF treated liposome preparation, smaller liposomes filled with iron particles can be found (Figure 1b, Panel IV). Almost no individual iron particles are on the larger remaining liposomes. In TEM images, when both ASMase and AMF are used, there are small iron-containing vesicles inside and outside the liposomes. This finding could be explained by the invagination of the ceramide- and iron-containing microdomains assisted by AMF. 

Small-angle X-ray scattering provides ensemble-averaged structural details on a smaller length scale (Figure 1c and Figures S1–S3). A continuous increase of scattering at the lowest q range was observed, which indicates that objects are larger than 60 nm (≈2π/q_min_). The liposomes, 5 nm Fe_3_O_4_ nanoparticles in PBS, and their mixture were measured. The scattering representing pure liposomes shows a small maximum, which indicates that there are mainly uni-lamellar liposomes in the system. The nanoparticle solution shows significant aggregation, demonstrated by plotting the iron nanoparticle in a so-called Kratky plot (refer to Figure S2 of Supporting Material) that shows maxima, which correspond to a characteristic distance between particles of 10 nm, i.e., twice the expected diameter of nanoparticles that are well-packed in larger aggregates. The shape of the scattering intensity of the mixture contains the main characteristics of the NP solution curve. The position of maximum due to particle–particle interaction shifts to a smaller q, which indicates that the distance between nanoparticles increased from 10 to 11–12 nm. Moreover, the maximum due to liposomes becomes less pronounced, which can be interpreted as a bulge structure in agreement with TEM. The SAXS data show that the magnetite has a different behavior when it is alone in the aqueous medium than when it is in a liposomal aqueous medium, thereby confirming the association of iron nanoparticles with the liposomes. This finding also shows a change in the iron’s behavior when SMase is used to treat the liposomes. One potential explanation for this behavior is that there is agglomeration that occurs to the small iron particles in the liposomes, although there are additional events and agglomeration that occur after the SMase treatment when the phase separation starts to develop after ceramide formation. 

If we examine the heat generation of SMase-treated liposomes, we observe a small heat increase of approximately 0.7 kelvin in 20 min of treatment with 1.5 mT magnet in room temperature (RT) conditions (Figure 1e). This increase is surprisingly small to explain the dramatic occurrence of sudden dis-attachment of iron filled vesicles. When we used 30 mT treatments, we identified a heat increase of 2 kelvin in SMase treated liposomes but only 1.5 kelvin in liposomes without SMase treatment. This finding, together with the TEM and SAXS data, demonstrates the agglomeration of the iron particles on the liposomal membrane that is increased when SMase induced ceramide rich microdomains occur in the liposome surface, moderately increasing the heat dissipation but causing a dramatic shift in the membrane organization and the budding of the smaller iron filled vesicles from the liposomes. This also explains the increased leakiness of the liposomes and allows other properties as well as the increased attachment of the liposomal membrane to other lipid membranes. 

The SMase treatment combined with AMF treatment increases the cluster formation of iron nanoparticles confirmed by SAXS measurements (Figure 1d and Figures S2–S4), and this increase is associated with increased leakage of the payload from the liposomes (Figure 1c). The synergistic effect of the enzyme and the applied magnetic field causes a stronger and more selective release than the enzyme treatment achieves alone (Figure 1c and Figure S2). The SAXS data show minor effects of AMF and SMase treatment on the structure of nanoparticle aggregates. AMF and SMase treatment appear to lead to a shift of the maximum to a smaller q and the maximum becomes less pronounced, pointing to a decreased order of NPs and an increased distance between nanoparticles of up to 12 nm. If AMF and SMase are both applied, the maximum returns to the previous position/height and at the same time the slope at the lowest q increases, which implies further aggregation of nanoparticles. These encouraging biophysical in vitro experiments support the use of SM-liposomes loaded with ferric nanoparticles as drug delivery vehicles. 

The MESL system described in this study permits leakage at low energy AMF (i.e., energies that do not increase the tissue temperature over 40 °C). Our AMF device is equipped with a cooling system to avoid an increase in temperature. In animal experiments, the surface temperature was measured in the AMF treated animals, and no increase of the temperature was observed. We aimed to test the AMF effect on classical thermosensitive liposomes, Dipalmitoylphosphatidylcholine:1,2-distearoyl-sn-glycero-3-phosphocholine (DPPC:DSPC, 90:10) [28], with the same enzymatic conditions and AMF range employed for MESL. The results demonstrated that the strength of AMF was not sufficient to open the phosphatidylcholine (PC) and iron nanoparticle-containing thermosensitive liposomes after enzyme incubation, in opposition to similarly treated MESL liposomes. 

TEM images and SAXS experiments show the agglomeration effects of the iron nanoparticles on the surface of the lipid membrane after enzyme treatment and following phase separation and domain formation. The release of cisplatin through a semipermeable membrane was analyzed with a spectrophotometer. More importantly, the liposomes were preincubated with increasing dosage of SMase, and the release of the drug was analyzed after 24 h of dialysis. The liposomes were held inside the dialysis bag; thus, released material can be analyzed from dialysis media. The drug release was triggered with mild 1.5 mT AMF of 20 min, and the subsequent leakage was analyzed (Figure 1c). 

The study demonstrates that even mild SMase activities are sufficient to assist in the release process when AMF is also used. The SMase-AMF combination was able to induce the leakage with enzyme concentrations over 100-fold lower (<0.01 U) than with the pure SMase-dependent method. 

The leakage assays indicate that the release occurs and demonstrate the synergistic effect of SMase and AMF (Figure 1c). 

### 2.2. Cell Studies

ASMase-induced ceramide accumulation, commonly observed in inflammation and stressed cancer tissues, represents an important marker of stress and apoptosis. Therefore, endogenous SMase can be used to make SM-containing nanoparticles change their membrane state from homogeneous to more heterogeneous through the lipid phase separation. Thus, it also makes them dose-responsive to environmental changes and ultimately leak their content in the proximity of stressed cells [22,29]. This feature makes sphingolipid liposomal nanoparticles and sphingolipid-coated nanoparticles interesting candidates for tailored drug release. There is no lipid phase separation-based sensor or delivery system at present, even though the ASMase based lipid phase separation has been regarded as one of the key elements in apoptotic cell stress. 

Oral squamous cell carcinoma (OSCC) consistently ranks as one of the top 10 common cancers worldwide, and the mortality of the recurrent disease is high. Cisplatin-loaded SM-liposomes were chosen because, in our cancer model, the head and neck carcinoma (squamous cell carcinoma, SCC), cisplatin is among the most common drugs used in adjuvant and neo-adjuvant therapies of advanced SCC [30,31]. We have previously shown that irradiation induces ASMase secretion in SCC-9 cells [32]. SCC-9 cells responded to the strong radiation stress through a route that leads to ASMase secretion on the outside of the cell membrane. We aimed to show the importance of SMase and AMF in the release 

The best timing for the external magnetic switch is when the liposome has reached the disease site where the secreted ASMase can act on the liposome. Overall, the non-pegylated liposomes have a short or intermediate lifetime in the blood circulation. Thus, the maximal targeting of the liposomes happens quickly in 30–120 min. We decided to strike magnetically to the liposomes when an excess of the target liposomes occupy the site. While the iron particles tend to agglomerate after phase transition following SMase treatment, AMF can be triggered after the exposure to SMase. Low magnetic field strengths allow the opening of the liposomes only in the place of SMase altered liposomes. In this setup, SMase converts liquid phase liposome towards more solid–liquid interface liposomes in which membrane-bound small iron nanoparticles can agglomerate to the solid microdomains. This finding creates an opportunity to further manipulate the activated liposomes with an alternating magnetic field (video S1). The change in lipid composition, domain formation, and iron particle agglomeration ultimately leads to the liposome rupturing and releasing of the payload (refer to video S1). 

**Figure 2:**
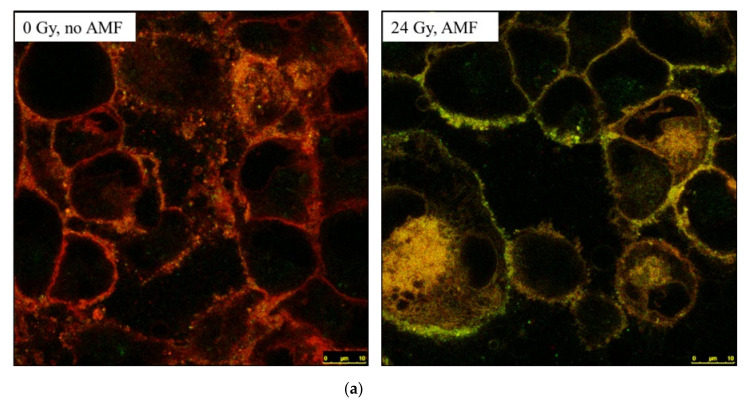

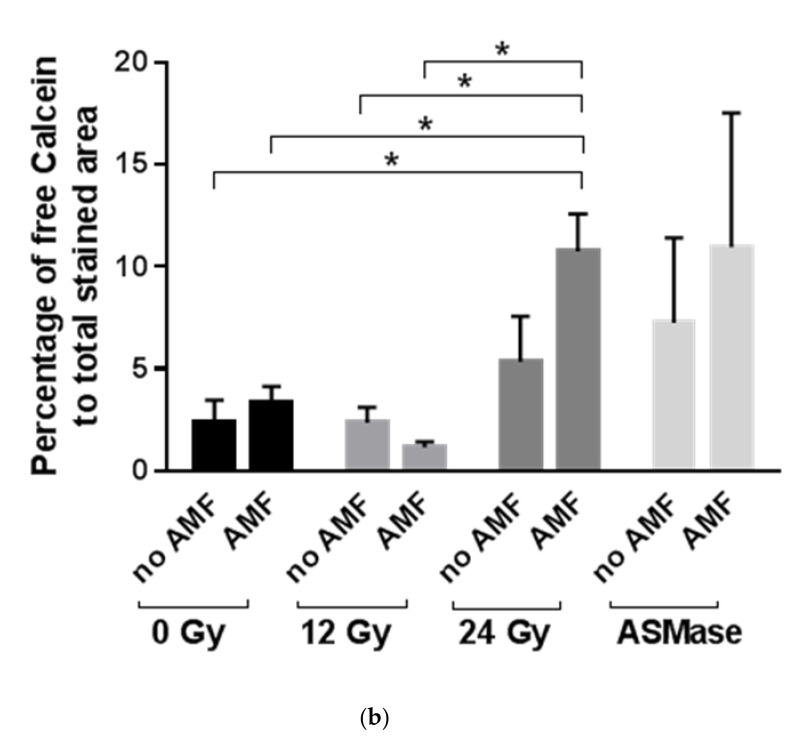

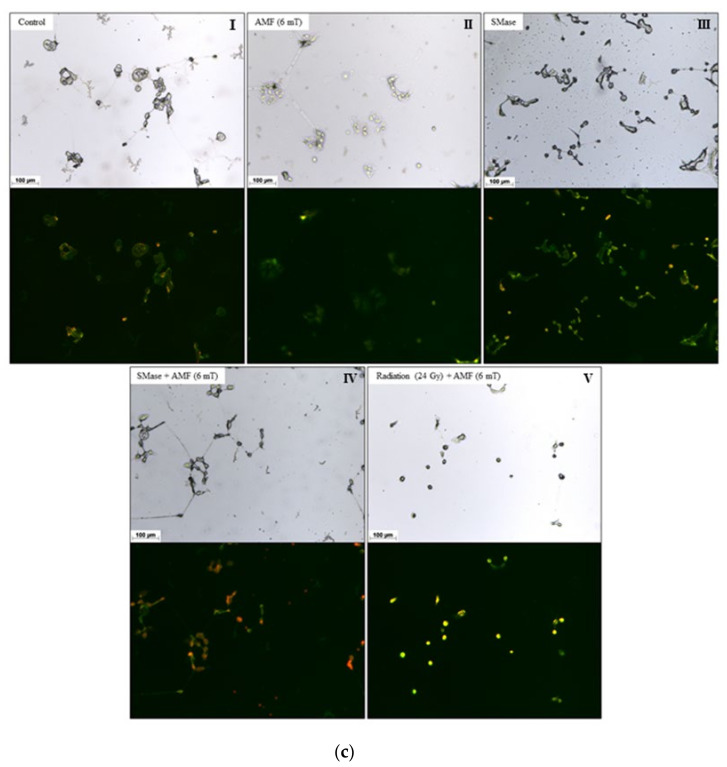
SCC cell images were observed by confocal microscope (63×). (**a**) Cellular interaction of SCC cells with the fluorescently (TeXas-Red PE) labeled SM-liposomes loaded with ferric nanoparticles and calcein. After the addition of the liposomes, the cells were exposed to SMase, AMF, or combined treatments. After the treatment, cells were washed and fixed with 4% PFA. Control cells that had liposomes without extra treatment (panel (I)) were observed with confocal microscopy and combined green (calcein drug mimic) and red (Texas Red liposomes) fluorescent images. Confocal image of the SCC cells after liposomes containing o,8-PE on the lipid membrane, and calcein as a payload. In the images, red-orange shows the liposome attachment to the cells after heavy 24 Gy radiation and AMF and nontreated control. (**b**) Quantification of calcein from the confocal images of the cells to show cell uptake of liposomal calcein cargo after induction of SMase by the stressed SCC cells as units/100,000 cells. Images were taken with a confocal microscope, and quantification of the freed payload was measured by calcein signal in the cells. SMase 0.4 U/mL was incubated 30 min before measurement, and in the AMF treatment, 6 mT 20 min was used. Different radiation times were used to determine the effect of spontaneous SMase after radiation stress and AMF in the release of liposomes. (**c**) Cellular interaction of SCC cells with the fluorescently (TeXas-Red PE) labeled SM-liposomes loaded with ferric nanoparticles and calcein. After adding the liposomes, the cells were exposed to SMase, AMF, or combined treatments. After the treatment, cells were washed and fixed with 4% PFA. Control cells that had liposomes without extra treatment (panel (I)) were observed with light microscopy and combined green (calcein drug mimic) and red (Texas Red liposomes) fluorescent images. Panel (II) presents cells that were treated with AMF (6 mT) after liposome addition, and Panel (III) presents cells treated only with sphingomyelinase (0.4 U/mL) after liposome addition. Panel (IV) presents combined treatments of SMase (0.4 U/mL) and AMF (6 mT). Panel (V) shows cells treated first with 24 Gy radiation, followed by the addition of liposomes and AMF treatment.

To confirm the interaction of SCC cells with liposomes and study the subsequent payload release triggered by the synergistic effect of SMase and AMF we conducted cell studies. Fluorescently labeled SM-liposomes loaded with calcein were applied to SCC cells (Figure 2a,b). While cisplatin itself and its release are very difficult to track in cell experiments, we used calcein-loaded vesicles as a model of encapsulated drugs. Calcein-loaded and Texas red membrane labeled liposomes served as an excellent fluorescent pair to conduct experiments with cells under confocal microscopy. We obtained confocal images, tracked the calcein fluorescence that was used as a payload, and compared it to the Texas Red labeled to phosphatidylethanolamine lipid (Texas Red™ 1,2-Dihexadecanoyl-sn-Glycero-3-Phosphoethanolamine, Texas red-PE) (used as a membrane stain in the liposomes) fluorescence in the similar cell settings (treatment time and environment). We observed a difference in the colocalization of the calcein compared to Texas Red after irradiation, AMF, and nontreated cells. Whereas Texas Red shows much higher signal intensity on the cellular borders than on the inside of the cells suggesting an altered internalization behavior, calcein is highly spread on the inside of the cells (see 24 Gy, with AMF (right)). Overall, the appearance of orange regions with red and green overlapping (Texas Red and calcein colocalized) decreases, shifting to green signals dominating. 

This effect is even more intense in fluorescence microscopy studies with an additional AMF treatment. After the addition of the liposomes, the cells were exposed to radiation-induced ASMase, SMase, AMF, or combined treatments, and after the treatment, cells were washed and fixed with 4% paraformaldehyde (PFA). Control cells that had liposomes without extra treatments (Figure 2c panel I) were kept in RT in the same conditions (time and environment as well as microscopy settings) as the treated cells. All cells were observed with light microscopy and combined green (calcein, drug mimic) and red (Texas Red liposomes) fluorescent images. All the changes in fluorescence intensity or the ratio of Texas Red and calcein signal reflect directly to the difference of the concentrations and the localization of the fluorophores. The cell experiments showed that the calcein encapsulated in the liposome lumen was not able to escape by the AMF or SMase treatment alone (Figure 2c, panels II and III (respectively)). Panel II presents cells that were treated with AMF after liposome addition. These panels show some liposomes homing to the cells with the signal colocalizing from calcein and the liposome marker. Panel III presents cells treated only with sphingomyelinase. In the SMase treated liposome image, we observe increased calcein uptake and some binding of liposomes to the cells, which can be explained by the tendency of ceramide to increase liposomes’ aggregation to the other membranes and thus also to the cells. Panel IV presents the combined treatments of SMase and AMF, and from the image, we can see the increased binding of the carrier to the cell and a decreased amount of calcein signal. It seems that the liposomes can associate with the cells. It has previously been shown that cationic liposomes can target angiogenic endothelial cells in tumors [33]. The results show strong binding of liposome marker to the cell surface but no internalization of calcein. This result is understandable, as the cell washes have been able to wash away all rapidly released calcein drug mimic. Panel V presents cells treated first with 24 Gy radiation, followed by the addition of liposomes and AMF treatment. The cells after 24 Gy radiation are damaged, and the morphology of the cells is altered. There are several articles that confirm that radiation will lead to substantial apoptosis and cause immediate cell damage and ASMase release [32]. Surface bound ASMase causes internalization of ceramide-rich domains and facilitates cell uptake. Overall, cells in all images (Figure 2c) are not looking healthy. This is due to the treatments done in non-optimal cell conditions: during the radiation and AMF/SMase treatments, the cells were kept out of the incubator and at room temperature. The control cells were treated similarly, as well. 

Our results show that only when ASMase was combined with AMF, calcein is released outside the liposomes and without being internalized by the cells. This finding suggests that at least with SCC cells, the MESL system can bring the drug to the cells; however, it depends on the drug whether it will be internalized by the cells.

### 2.3. Animal Studies

**Figure 3:**
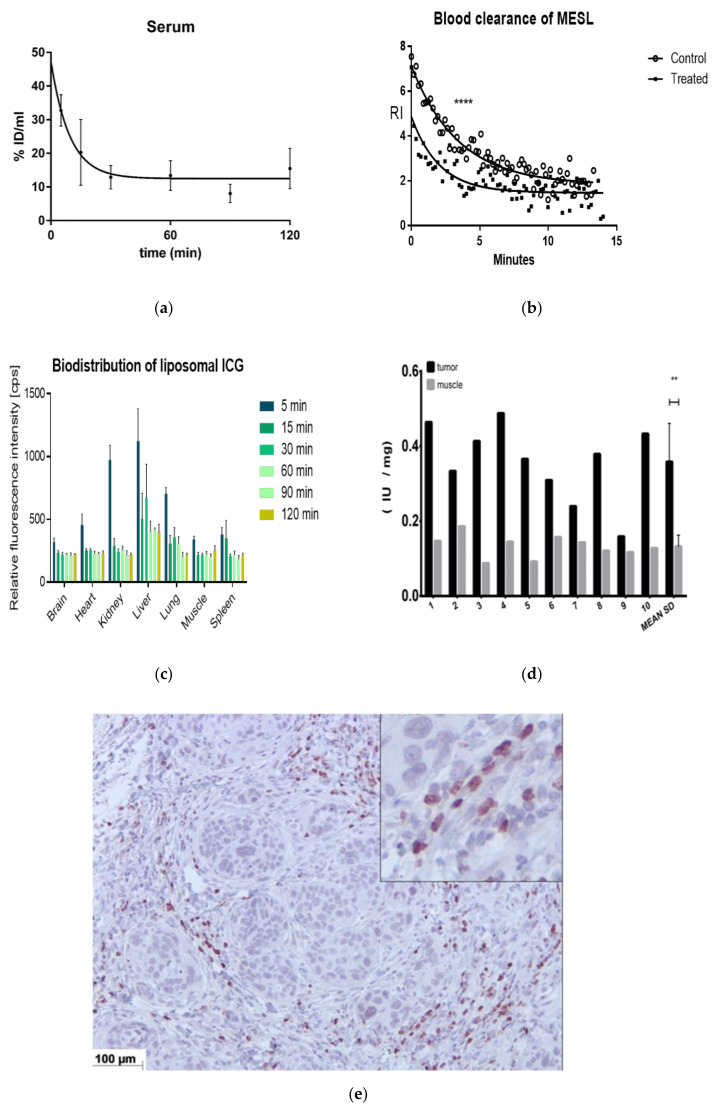

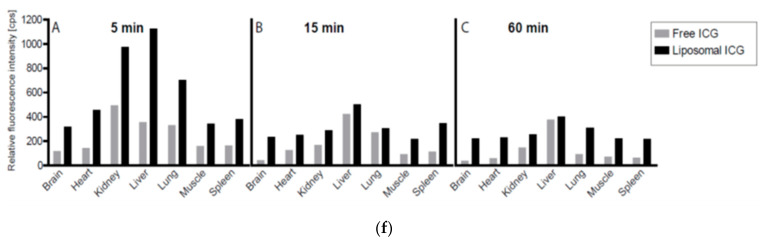
Characterization in vivo. (**a**) Blood clearance of the MESL liposomal particles loaded with ICG. The image shows a plot of ICG signal from the blood represented as %ID/mL, as determined by the absorbance at 800 nm at 5 (*n* = 3), 15 (*n* = 4), 30 (*n* = 6), 60 (*n* = 6), 90 (*n* = 3), and 120 (*n* = 4) min after intravenous injection. Each bar represents mean ± SEM. (**b**) Dynamic blood kinetics determined using photoacoustic imaging of the mouse femoral artery. RI in y-axes represents relative intensity for fluorescence. The injected amount was the same as well as c max in blood. Both nontreated SM- liposomes (●) (DSPC:SM:DOTAP:chol (20:30:20:30) mol/mol, loaded with Fe NPs and ICG)), as a control, and SM-liposomes pretreated with SMase (0.4 U), and AMF (5 min 2 mT, 100 kHz) (■) were injected in the mouse tail vein and followed by imaging femoral artery for 15 min (*p*-value 0.0001, *t*-test two-tailed unpaired). (**c**) Biodistribution of ICG filled MESL liposomal nanoparticles for the first 2 h after IV injection. Fluorescence images were acquired after 5 (*n* = 3), 15 (*n* = 4), 30 (*n* = 6), 60 (*n* = 6), 90 (*n* = 3), and 120 (*n* = 4) min. Each bar represents the mean ± SEM. (**d**) ASMase activities analyzed with Amplex Red reagent-based assay in human SCC-9 tumor xenografts in mice. Muscle tissue was analyzed as control tissue. The activity was assessed immediately after mouse decapitation and dissection and was normalized by the weight of the tissue sample. In the average columns, both bars represent mean ± SEM. ** represent *t*-test results smaller than 0.005. (**e**) Histological staining of ASMase from SCC tumor xenograft slices. (**f**) We compared biodistribution of the liposomal ICG to Free ICG, which shows the liposomal ICG to be in circulation for longer. This result was also observed in the blood kinetic studies.

In addition to the physicochemical- and in vitro-cell studies, the delivery system was tested in animals. The pharmacological parameters were analyzed in healthy mice. The clearance of the MESL delivery system from the blood circulation was studied after injection of ICG-MESL via the tail vein by analyzing the ICG absorbance from ex vivo blood samples (Figure 3a) and by the in vivo photoacoustic imaging directly from a mouse femoral artery (Figure 3b). Moreover, the ICG biodistribution was analyzed from the major organs (Figure 3c). The dynamic liposomal ICG imaging performed by photoacoustic imaging suggests the presence of two components in the blood after AMF and SMase treatment: one fast clearing ICG component and one slowly clearing, presumably liposomal, ICG component. Photoacoustics were more suitable for the analysis of the early time points, whereas ICG absorbance from ex-vivo blood samples and biodistribution were preferable for time points after the first 15 min. The biodistribution indicates that the main secretion route for ICG is hepatobiliary. Blood clearance was obtained by post-mortem heart puncture, and the ICG level was determined by absorbance of the ICG; the total blood value was then estimated (Figure 3a). We also performed the photoacoustic analysis by imaging the leg aortic vein during the injection and then dynamically evaluating the clearance in time function (Figure 3b). From this measurement we can obtain the plasma concentration right at the time of the injection and also dynamic clearance data. These two components enable a two-compartment model to be performed. This is an important feature of the nanoparticle: from more rapid ICG clearance when SMase and AMF were used in the pretreatment of the liposomes, it can be concluded that there were leakages of ICG from liposomes and the leaked ICG from liposomes is cleared faster than liposome bound ICG (Figure 3f). It is generally observed that the liposome or nanoparticle-bound drugs are cleared slower than free drugs when nanoparticles are over 10 nm in size, because nanoparticles can protect drugs from rapid kidney clearance. The large nanoparticles cannot be cleared through the kidneys because they are larger than the kidney-threshold. Liposome accumulation to Kupffer cells is one of the most known mechanisms of liposome liver accumulation [34]. However, there are also other cell types that can contribute to liposome uptake in the liver like hepatic antigen presenting cells, liver sinusoidal endothelial cells, and hepatocytes. Together they form a tolerogenic environment that allows efficient processing of material facing liver [35,36]. We did not study the hepatobiliary secretion of the liposomal phospholipids, but we did observe the ICG and ICG metabolites homing to and clearing from the liver (Figure 3c). During blood circulation, immune cells can internalize the liposomes and then transport them to different immunologically active sites [37,38].

It is essential to test that the cells and tissues of interest provide sufficient amounts of SMase in biologically relevant conditions that the MESL could work adequately in a biological environment. The ASMase activity in the tumor area was determined with an optical ASMase activity kit that indicated higher ASMase activity in the tumor tissue than in the muscle tissue (Figure 3d). The SMase levels in SCC tumors grown in mice, and the front leg muscle ASMase levels were simultaneously analyzed to assess the background levels. 

We have demonstrated that the SCC-9 cell line expresses secreted ASMase to its surroundings when it is stressed by radiation [32]: it has been shown that endothelial cells secrete ASMase in lower radiation [39]; however, it has also been reported that ASMase can be secreted by immune system cells [40]. Thus, it is highly likely that several cells in the stroma are involved in ASMase secretion. Our immunohistological samples from human SCC tumor cells grown in mice show the ASMase location in the tumors (Figure 3e). ASMase can be found both on the outer borderline of the tumor region and in the stroma. In the histopathological slices, the cells in the stroma appear to be strongly ASMase-positive, as predicted. 

### 2.4. Therapy Study

**Figure 4:**
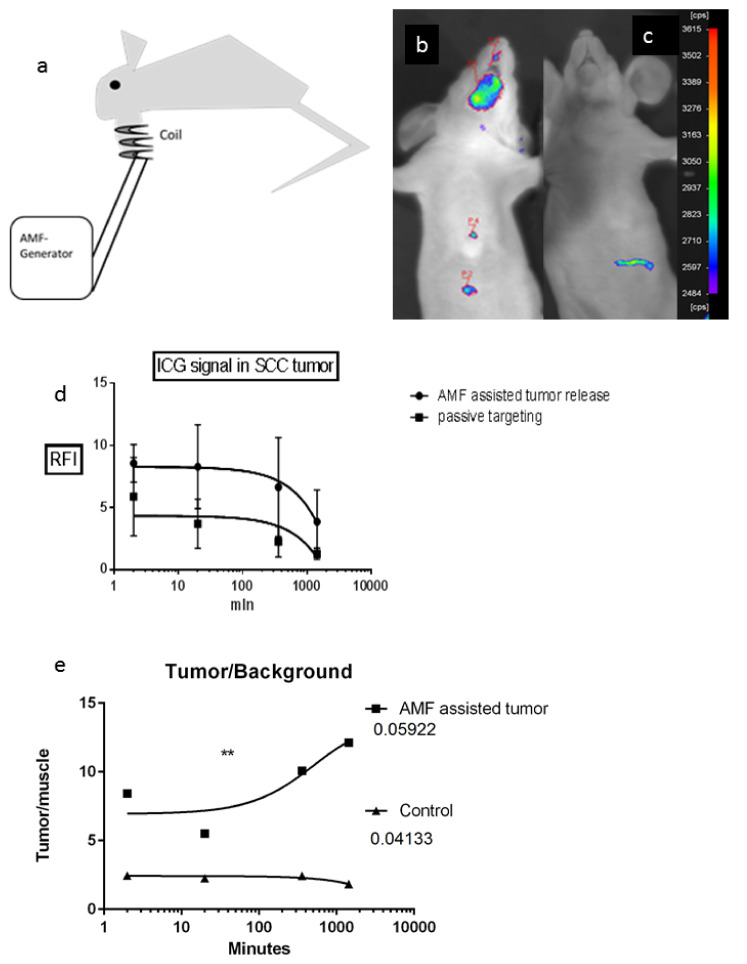

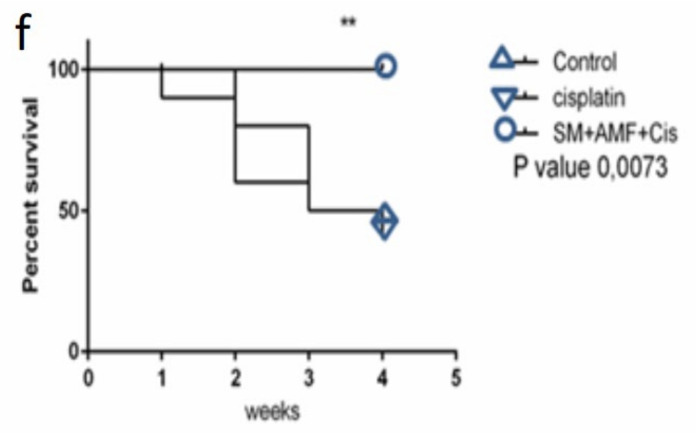
Targeting studies in vivo. (**a**) Schematic drawing of the experimental setup used in AMF experiments for treatment of oral squamous cell carcinoma (SCC). The top of the coil is placed near the tumor, and the tumor is exposed to AMF treatment. (**b**) Visualization of the ICG imaging marker delivered by SM-liposomes (MESL) with ferric nanoparticles in an orthotopic mouse model of SCC9 oral squamous cell carcinoma. The representative mice were imaged after 6 h of IV injection of ICG that contained MESL. After tail vein injection of MESL, mice were treated with AMF (20 min, 1.5 mT, 100 kHz). (**c**) The control mice received an IV injection of MESL with ICG without AMF treatment. The representative mouse was imaged 6 h after injection. (**d**) Longitudinal comparison of tumor uptakes of ICG delivered by MESL delivery system treated with (●) or without (■) AMF treatment. Quantification of relative fluorescent signal (RFI) from ICG signal in SCC-9 human xenograft bearing nude mice was imaged by Night OWL imaging system. The study was conducted after the injection of ICG and iron nanoparticles containing SM-liposomes. Immediately after injection of liposomes, the tumor region was treated with 1.5 mT AMF (20 min) vs. the control group that was not subject to AMF. Each bar represents the mean ± SEM. (**e**) Longitudinal comparison of in vivo tumors to background fluorescence ratio of ICG containing MESL with AMF treatment (■) or the same liposomes without AMF treatment (▲). Tumor signals were derived from the SCC tumor, and the muscle tissue from the front leg was used as the background tissue. (*t*-test *p*-value 0.0029). (**f**) Kaplan–Meier presentation of the survival study of SCC-9 tumor-bearing orthotopic xenograft mice. Mice were treated with IV injections of ICG, cisplatin (∆) (120 μL 0.65 mg/mL equivalent to 12 mg/m^2^) or liposomal cisplatin formulations (°) (120 μL 0,65 mg/mL equivalent 12 mg/m^2^) once per week for 4 consecutive weeks. *p* value 0.0073 treated versus control.

In a final proof of principle experiment, the nanoparticle system was tested for therapy applications given the increased survival benefit and positive changes in the tumor compared to the control delivery system and free drug. Due to the observation that orthotopic SCC-9 tumors grown in nude mice exhibit several-fold higher ASMase levels and activity than in the muscle tissue in the same animals (Figure 3d,e), we used an experimental setup as depicted in Figure 4a to test whether the MESL delivery together with AMF results in an increased uptake in the site of disease. ICG carrying MESL were injected into the tail vein of SCC-tumor-bearing mice. The ICG signal increase by MESL is clearly visible in the tumor area under the chin of the AMF treated mice (Figure 4b) compared to the non-AMF treated control mice (Figure 4c) or animals treated with control liposomes, where SM lipid was replaced with PC-lipid (Figure 4c). Longitudinal imaging of ICG-MESL treated tumors indicates an increased signal in the tumors treated with AMF compared with the non-AMF treated tumors throughout the imaging time, leading to a higher area under the curve (AUC) (Figure 4d). The signal to background ratio was calculated by comparing the tumor signal to fluorescence in the mouse front leg muscle. There was a significant increase in the tumor to background ratio of the ICG signal in ICG-MESL, with the AMF treatment group reaching 12-fold at the end of the study (Figure 4e). The ICG signal carried by MESL without AMF treatment did not increase the ratio in the first 24 h. 

As an animal model for therapy, SCC mouth tumors were chosen. These orthotopic tumors are convenient for optical imaging. We aimed to start with a relatively low treatment dose to control whether the new formulation has an increased effect without causing new adverse side effects. Thus, we used the Shannon Reagan-Shaw calculation ((Animal dose mg/kg * animal Km (3))/human Km (37)) to reach animal doses similar to the dosing of cisplatin in human patients. 

We used the dose that equals the lowest recommended repeated dose, currently 20 mg/m^2^, used in combination with radiation [41]. Our cisplatin concentrations in animals were ≈5 mg/kg, which correlates well after the Shannon Reagan-Shaw conversion with the 0.5 mg/kg concentration equaling a 20 mg/m^2^ dose in the human reference. The survival studies confirm that the cisplatin-AMF-MESL treated animals survived longer than their liposomal or free cisplatin (same dose) treated peers (Figure 4f).

## 3. Discussion

This work presented a novel technique for controlled release using magneto-enzymatically opened iron nanoparticle-containing sphingomyelin liposomes. In this nanolipid delivery system, ICG and cisplatin were used for imaging and therapy. This delivery system offers a novel solution for the clinical problem of releasing the drug from the targeted delivery system. The rationale for lipid selection was that 30% of cholesterol is needed to increase the liposomes’ stability, and the SM-lipid amount of 30% was optimized for enzymatic opening and encapsulation efficiency. DSPC is the backbone lipid for the liposomes, and it was chosen while the lipid is generally not included in the thermosensitive PC-lipid category. 1,2-dioleoyl-3-trimethylammonium-propane (DOTAP) is added to maintain the liposome surface charge other than zero to avoid liposomes’ aggregation when stored. We decided to have a cationic surface charge on the liposomes because we wanted the liposomes to be cleared out fast from the body after local AMF instead of the long circulation EPR targeting strategy. The general strategy was to use components that have previously been approved for clinical use. 

When biocompatible magnetic nanoparticles are placed in the bilayer, the membrane can be destabilized if subjected to a localized, alternating magnetic field. Magnetic nanoparticles produce heat in an alternating magnetic field. Their heating capacity is based on size and shape. In general, iron nanoparticles over 30 nm in diameter are suitable to heat generators, whereas iron nanoparticles below 10 nm have a low ability to conduct magnetic energy to the heat [25]. Recently, it has been shown that small iron nanoparticles, when agglomerated, can change their propensity and generate more heat [42,43]. Small 5 nm magnetic nanoparticles we use in MESL do not dissipate heat in an alternating magnetic field. Small nanoparticles can cluster in the ceramide lipid microdomains produced by SMase enzyme on liposomes. When iron particles agglomerate, they can bring energy to the liposomes. We provide proof that this agglomeration indeed could be the case based on our TEM images (Figure 1b). When heat-generating iron nanoparticles are associated with liposomes, they can lead to liposomal disruption and release of their content [43,44,45,46]. Our concept in which a membrane status changes and membrane reordering causes accumulation of particles and thus change the energy dissipation properties is new. One should conclude from SAXS data that enzyme treatment leads to additional aggregates of iron particles. TEM images and SAXS experiments together precisely show the agglomeration effects of the iron nanoparticles on the surface of the lipid membrane after enzyme treatment and following probable phase separation and domain formation. The maximum shows the mean distance between iron particles is approximately 120 Å. It appears that the maximum is higher, which suggests stronger repulsion between iron particles for Indocyanine green liposomes than for cisplatin liposomes. The apparent radius of gyration also increases from 215 to 239 Å. AMF treatment leads to a higher aggregation of iron nanoparticles.

We and others have previously shown that SMase causes a dramatic membrane disruption in the liposomes, where liposomal sphingomyelin is converted to ceramide, resulting in ceramide rich domains [21]. The formed clusters and small iron consisting vesicles observed here interestingly resemble the domain formation previously observed in ceramide consisting of liposomes [19]. The change from SM/Cholesterol/PC/DOTAP liposome to SM/Ceramide/Cholesterol/PC/DOTAP liposomes changes likely the phase transition temperature. Our formulation permits an enhanced release rate; however, it does not produce this release via temperature increase but by a phase transition of the membrane. In vitro and in vivo temperature remains stable within 1 °C when field strengths under 6mT are used. The leakage experiments were done in RT. In mouse model the experiments were done below the normal phase transition temperature, and no heating of animals was observed in these field strengths. The external energy brought to the system by magnetism most likely causes the lipid bilayer to change the state of lipid film via lateral movement and phase transition, leading to increased leakiness of the membrane.

The enzyme effect and AMF form a molecular switch mechanism that enables a targeted release in ASMase-involved tissues. In this study, we performed several biophysical in vitro assays to study how the MESL formulation’s design having iron nanoparticles embedded in the lipid surface operates in different environments and stimuli by analyzing release. The presented SM containing liposome carrier features a specific cell stress controllable release mechanism, enabling the liposome’s rupture in the active SMase location aided by the magnetic field. An alternating magnetic field can be applied on the accumulated carrier system site and used to open liposomes if a sufficient amount of SMase is expressed. The presented delivery and release are only initiated if the action of SMase already modifies SM-liposomes during a clinical condition, not if only a low magnetic field AMF (field strength <6 mT) is applied. 

Our proof of concept imaging data powerfully shows the synergy in the release system combining ASMase enzyme function with the magnetic switch. Ex vivo human studies demonstrate that the ASMase levels are significantly elevated in individual cancer patients at the early stages of cancer [47,48]. ASMase activity of the tumor and stroma, particularly the interplay of tumor cells and platelets, is demonstrated to be crucial for tumor metastases [49,50]. 

In several other pathological conditions, such as rheumatoid arthritis and colitis, increased SMase levels have been observed [50,51]. To our knowledge, the presented system is the only described method to date that can deliver drugs and imaging markers efficiently and control the site of tumors and inflammation by upregulated ASMase activity. 

One way to intensify the drug release locally in the desired location is to use thermosensitive liposomes together with an internal heat source or high intensity focused ultrasound (HIFU) [43,46]. The use of hyperthermia caused by AMF as a modulator has also previously been studied [42,52]. However, the used liposomal nanosystems are nonselective, and high magnetic fields or heating are required, causing local damages in the site of treatment. Thermosensitive liposomes used in these systems tend to leak when they are in the blood circulation prematurely. Therefore, a method for efficient and controlled release without extreme heating conditions is required. In contrast, our method enables the usage of a lower field of AMF, which is safe without the classical side effects of AMF-assisted heat enthalpy therapy or heat-based liposomal release [53,54,55]. Magnetic hyperthermia (MHT) has been employed for several decades; however, it was only recently approved as a clinical-grade standalone therapy for glioblastoma and prostate cancer [56,57]. Our H0 ≈ 1.6 kA/m and frequency f = 100 kHz are substantially below these accepted fields. 

In head and neck carcinomas, the primary tumor size and sentinel biopsy and neck dissections are commonly used to evaluate a potential need for adjuvant oncological therapy to prevent metastasis formation. However, these variables are relatively coarse predictors, particularly in cases of small and mid-range sized tumors. Orthotopic tongue tumors in mice, implanted by our head and neck surgeons, were chosen as a model for this optical imaging study, while tongue cancer is a viable choice in the following possible clinical trials. Imaging is possible because the epithelial layer is thin, and the tumors locate close to the epithelium. Cisplatin is commonly used as a drug of choice in SCC’s therapeutic strategy after surgery combined with radiation. The ICG imaging indicated substantially more ICG signals in the AMF treated tumors than in the non-AMF treated tumors or free ICG. These findings show that MESL liposomes with cisplatin and ICG enable better accumulation of the dye and the anticancer drug, allowing efficient imaging and a better medicine homing and thus better therapy outcome due to the better bioavailability of the drug (Figure 4d) [58,59,60].

The ICG fluorescence in MESL can also be used in an intraoperative setting and an operating microscope with an infrared fluorescent imaging capacity. Surgeons can detect cell stress, potential micrometastases, and tumor borders in the intraoperative environment. Additionally, photoacoustic imaging is a new method that offers in-depth visualization possibilities, up to 10 cm, of fluorescent nano-bio sensors using ICG [61]. Currently, a robotic DaVinci system equipped with a camera with fluorescence filters suited for ICG imaging is available [62]. Imaging of cell stress combined with the possibility of action enables more nuanced individual options in the future for treating tumors throughout the tumor progression. 

The system’s translation is possible in the future because all parts of our delivery system are currently being used in pharmaceutical formulations, and the AMF dose is generally regarded as harmless. However, we acknowledge the need for testing the system with several animal species to reach human therapy studies. Further studies are needed to investigate and optimize all possibilities that this two-component system can provide for clinical drug delivery and imaging.

## 4. Materials and Methods 

### 4.1. Liposome Formulations

Lipids: 1,2-distearoyl-*sn*-glycero-3-phosphocholine (DSPC), 1,2-dioleoyl-3-trimethylammonium-propane (DOTAP), cholesterol, and Sphingomyelin (SM) from egg were purchased from Avanti Polar Lipids (Alabaster, AL, USA), sphingomyelinase (SMase) from *B. cereus* was from Sigma Aldrich (Taufkirchen, Germany), and carboxyl-coated 5 nm Fe_3_O_4_ with catechol-PEG(400)-COOH nanoparticles were obtained from AC Diagnostics Inc. (Fayetteville, AR, USA).Cisplatin (Sigma, Neustadt, Germany) or indocyanine green (ICG) (Sigma, Neustadt, Germany) -loaded liposomes were made using a lipid mixture of DSPC/cholesterol/SM/DOTAP (20:30:30:20 mol%, respectively) that consisted of a total of 20 µmole of lipids. Lipids stored in chloroform were pipetted to a round-bottomed flask, dried under nitrogen, and lyophilized for at least 2 h to remove trace amounts of chloroform. Small iron nanoparticles were inserted into the SM-liposomes to predispose liposomes to AMF (Figure 1a). Carboxyl-functionalized Fe_3_O_4_ nanoparticles (NPs) with an average diameter of 5 nm were used to provide the liposomes with magnetic responsiveness through increased Brownian motion of the molecules in the lipid film. The small size iron-oxide nanoparticles have been reported to spontaneously incorporate into the lipid films [63]. Lipids were allowed to hydrate for 30 min in 60 °C PBS that contained cisplatin (9 mM (0.5 mg/mL)) or ICG (1 mg/mL) and nanoparticle iron at a final concentration of 1.2, 0.6, 0.3, or 0.15 mg/mL after PD-10 purification. The liposome solution was freeze–thawed three times. Extrusion was performed 11 times through a 100 nm polycarbon membrane using a small volume extruder, or unilamellar liposomes were prepared using a needle tip sonicator (4 × 15 s low energy on ice). Liposomes were purified from the unbound compounds using a PD-10 column. Liposomes were controlled by liposome size measurements using dynamic light scattering (DLS). Reorganization of nanoparticles in the liposome system as an effect of AFM and SMase treatment was visualized by small angle X-ray scattering. 

### 4.2. Transmission Electron Microscope (TEM) Imaging

Negative staining room temperature transmission electron microscopy (TEM) was performed as previously described [64]. Briefly, 5 µL of liposome suspension was added to previously negative glow discharged holy carbon grids (Science Service, Munich, Germany) and incubated for 10 s. After removing the excess sample with filter paper, liposomes were stained twice with 5 µL of a half-saturated uranyl acetate solution and air-dried. Samples were then transferred into a JEOL 1400Plus TEM, and images were obtained at 100 kV acceleration voltage on a TVIPS F416 4 kx4 k camera (Tietz Video and Image Processing Systems, Munich, Germany). 

### 4.3. Small Angle X-ray Scattering (SAXS)

The SAXS measurements were performed with a laboratory instrument (Nanostar, Bruker AXS GmbH, Karlsruhe, Germany) using the wavelength of the Cu Kα line. The accessible q range was 0.009–0.23 Å^−1^. Samples were filled into glass capillaries of 2 mm diameter. The raw scattering data were corrected for the background from the solvent, black current of the detector, and shadow scattering of beamstop and converted to absolute units using the scattering of pure water measured at 20 °C (program SuperSAXS, Prof. C. L. P. de Oliveira and Prof. J. S. Pedersen). The ASMase and AMF-based iron aggregate formation is in good agreement with the TEM data.

The investigated interval of scattering vector q was from 0.009 to 0.23 Å^−1^, and it provides a possibility to obtain a full structure analysis of objects in length scale (R) from 10 to 300 Å. We did not observe signs of plato in all scattering curves at the lowest q range, which indicates that objects are larger than 300 Å. In the present experiments, we can obtain information regarding the internal structure of these objects or their interface. Pure liposomes show a very low scattering signal, and the data quality is not sufficient to perform a quantitative analysis. We can conclude from the absence of brag peaks in the intermediate q range that there is no multilayer vesicle formation. 

For cisplatin liposomes with iron nanoparticles, we observe a substantially stronger signal due to the higher X-ray contrast (≈ 50 times) for iron nanoparticles. Moreover, the objects are larger than the maximal detectable size of applied laboratory instrument (larger than R≈ 300 Å), and the internal organization of objects is observed via SAXS. The mean distance is approximately 100 Å. The shift of the maximum for iron nanoparticles incorporated into liposomes and after enzyme and AMF treatment (Figure S2) can indicate that the distance between particles slightly changes. Model independent analysis (indirect Fourier transformation) [65,66] also indicates the increase of the apparent radius of gyration from 205 to 229 Å (should be considered as low limit) and a parameter of the scattering at zero angle (I(0)) increases from 16 to 25 cm^−1^. It can indicate that the concentration of *n* particles and/or volume increases. I(0) is connected with the concentration and volume via I(0) = nΔρ^2^V^2^; here, *n* is the concentration of iron particles, Δρ is the scattering contrast and V is the volume of iron particles. 

### 4.4. AMF Treatment

The in vitro studies presented in this work were performed using a commercial AMF device (DM100, nB nanoScale Biomagnetics, Zaragoza, Spain) with variable field amplitudes H from 4 to 24 kA/m (5–30 mT) and frequencies between *f* = 229 and *f* = 823 kHz. The AMF for these experiments was controlled using a DM100 machine (4 ≤ H ≤ 24 kA/m; 229 ≤ *f* ≤ 823 kHz). Different concentrations (0.15–1.2 mg/mL of Fe_3_O_4_ nanoparticles per 13 micro moles/mL lipids in PBS) and different sizes (5–20 nm) of Fe_3_O_4_ nanoparticles embedded in SM-liposomes loaded with ICG in a final volume of 750 µL were incubated for 10 min in 4–24 kA/m fields. Both the leakage of the liposomes and the magnetic hyperthermia capability were assessed, and specific power absorption (SPA) values were analyzed.

The in vivo experiments and several in vitro experiments were performed at fixed conditions (1.5 mT and 100 kHz or 2.0 mT and 100 kHz) with a lab-made machine that included a water cooling coil embedded between copper coils built by our group. We measured the heating caused by our own coil by using an infrared thermometer which were used in cell studies and in vivo studies. We never saw higher increase than 0.7 kelvin with our mouse and biological studies. In vitro, AMF treatments were performed for 20 min for each sample containing 500 µL of liposomes previously described. If a sample was pretreated SMase, it was incubated for 30 min before applying the AMF treatment. After the treatments, the samples were inserted into a snakeskin dialysis bag (3.5 K MWCO with the 22 mm tubing dry diameter) and dialyzed for 24 h against PBS to analyze the released molecules. After 24 h incubation, both solutions inside and outside of the bag were analyzed by measuring the absorbance, and the leakage rate was calculated. 

### 4.5. Cell Experiments 

#### Fluorescence Microscopy

A total of 50,000 SCC cells were grown on glass slides overnight. The next day, the cells were subsequently exposed to fluorescently labeled liposomes DSPC:SM:DOTAP:chol, molar% 20:30:20:30, respectively, labeled with Texas Red^®^ DHPE (0.5 molar%, ThermoFisher) for 30 min. Liposomes were loaded with Fe_3_O_4_ nanoparticles (0.3 mg/mL) and calcein (10 µM) (Sigma, Neustadt, Germany). Cells were also treated with SMase (0.4 U/mL, 30 min) or radiated (24 Gy) and further exposed to a temperature-controlled AMF (6 mT, 20 min). Controls were solely treated with liposomes or also exposed to an AMF (6 mT, 20 Min) or SMase (0.4 U/mL, 30 min), respectively. The final treatment time and microscopy imaging time (endpoint at 30 min) was the same for all the cell groups and done in RT. After the treatments, the cells were washed three times with PBS in order to wash out free drug and liposomes that were not associated with the cells and fixed with 4% PFA. The fluorescence of calcein (496 nm excitation and 508 nm emission) and liposomal Texas Red (589 nm excitation and 615 nm emission) was imaged with a Zeiss Axio observer Z1 using AxioVision SE64 software or confocal laser scanning microscope Leica SP5 (Objective: HCX PL APO CS 63x/1.4 OIL).

### 4.6. Animal Experiments

#### 4.6.1. Biodistribution and Blood Clearance

Male and female albino mice (C57/BL6, 4–6 months, Source Charles River) were housed in a temperature and humidity controlled environment with a 12 h light-dark-cycle and access to food and water ad libitum. Mice were randomly divided into two groups: treatment (*n* = 22) and control (*n* = 13) groups. All experiments were carried out in accordance with the guidelines for Animal Care at the University of Kiel and have been approved by our local animal experimentation ethics committee {MELUR, ethic code: V 242-65884/2015 (88-6/15)}. The group size was estimated using biometric planning and was carried out with the aid of the statistics program G * Power 3.1.9.2 of the University of Düsseldorf (http://www.gpower.hhu.de/). F tests were performed with ANOVA. The group size was 10 animals/group.

#### 4.6.2. Immunohistological Staining

Tumor tissues were embedded in paraffin, and 3 µm slices were cut. The slices were deparaffinized and then demasked by cooking in a vacuum with citrate buffer pH 6.0 and blocked with peroxidase block followed by blocking with 10% nonfat dry milk. The sections were stained with AEC (Zytomed, Bargteheide, Germany) and counterstained with hemalum solution (Waldeck, Münster, Germany); for immunohistochemistry (IHC) analysis, the sections were stained with primary antibody specific for Acid sphingomyelinase (bs-6318R,Bioss Antibodies Inc., Woburn, MA, USA) and diluted 1/1000 in 10% milk, followed by HRP-polymer-anti-Rabbit (Zytomed, Bargteheide, Germany). The slides were attached with Pertex.

#### 4.6.3. Liposomal Nanoparticle Injection, Imaging, and Biodistribution

Animals were anesthetized with an intraperitoneal injection of 80 mg/kg ketamine (AVECO Pharmaceuticals, IA, USA) and 0.5 mg/kg medetomidine (Dorbene, Pfizer Deutschland GmbH, Berlin, Germany). All imaging agents were inserted via a tail vein injection. The treatment group received 150 μL of ICG-filled SM or PC liposomes corresponding to a total amount of 50 µg of enclosed ICG through the tail vein. The animal was exposed to AMF (20 min at 1.5 mT, 100 kHz). Thirty minutes after the injection, near-infrared imaging was performed in a NightOwl fluorescence imaging chamber (Berthold, Bad Wildbad, Germany). The control group received the same liposomal injection but no AMF treatment. The animals of the treatment group were euthanized 5 min (*n* = 3), 15 min (*n* = 4), 30 min (*n* = 6), 60 min (*n* = 6), 90 min (*n* = 3), or 120 min (*n* = 4) post-injection, followed by imaging of the organs. Images were created and analyzed with indiGO^TM^ Software (Berthold Technologies GmbH & Co. KG, Bad Wildbad, Germany). Individual organs were segmented by drawing regions of interest (ROIs); the fluorescent signal was determined in counts per second (cps). The average cps of all organs at one time point were summarized in a mean value that was then termed the relative fluorescence intensity (RFI). For the determination of the concentration of ICG-filled liposomal nanoparticles in the blood, the absorbance was measured at 800 nm with a SmartSpec Plus spectrophotometer (Bio-Rad Laboratories, Inc., Hercules, CA, USA). The concentration was evaluated using a standard curve prepared in vitro separately.

#### 4.6.4. In Vivo Tumor Imaging

In vivo imaging experiments were performed with 30 athymic female nude mice bearing an orthotopic SCC-9 carcinoma in the lower jaw region. The animals were anesthetized with ketamine (110 mg/kg)/medetomidine (0.5 mg/kg) during handling and imaging. Liposomes containing ICG fluorophores and Fe_3_O_4_ nanoparticles in a volume of 150 µL were administered via tail vein injection. The mice were imaged with the NightOWL camera, and image analysis was performed with indigo software. Moreover, the mice were imaged with a fluorescent tomography (FMT 2500, Perkin Elmer, Inc., Waltham, MA, USA). The mice were positioned so that the tumor was inside the magnetic coil of the AMF apparatus (Figure 4a). AMF (1.5 mT and 100 kHz) was applied to the tumor for 20 min. All mice were imaged after 1 h of the IV injection. A longitudinal imaging study was performed as previously described. The AMF treated, and nontreated animals were imaged after 0 min, 20 min, 6 h, and 24 h of the treatment.

#### 4.6.5. Survival Studies

In vivo imaging experiments were performed with 30 athymic female nude mice bearing an orthotopic SCC-9 carcinoma in the lower jaw region. The animals were anesthetized with ketamine (110 mg/kg)/medetomidine (0.5 mg/kg) during handling and imaging. The mice were assigned to the treatment or control groups for survival studies. The tumor volumes (mm^3^) were computed on ultrasound. The mice were treated with IV injections of ICG, cisplatin (120 μL 0.65 mg/mL equivalent 12 mg/m^2^) or liposomal cisplatin formulations (120 μL 0.65 mg/mL equivalent 12 mg/m^2^) once per week for four consecutive weeks. Weekly weights were recorded. At treatment termination, all mice underwent a repeat ultrasound to assess changes in the tumor volumes. Tumor volume ratios were computed by dividing the post-treatment by pretreatment (day 0) values for individual mice and as cohort averages. All mice were monitored daily, during, and following treatment and were euthanized for lethargy, hunched appearance, respiratory distress, or >15% body weight loss. 

#### 4.6.6. Photoacoustic Imaging

Photoacoustic imaging was performed using a custom-built real-time optoacoustic imaging system photoacoustic device (MSOT inVision 256-TF, iThera Medical, Munich, Germany) equipped with an external 3D probe. The probe consisted of a 3-dimensional array of 384 spherically focused transducer elements, each with a center frequency of 2.5 MHz (45% bandwidth, 4 cm radius of curvature, and maximal spatial resolution of 310 µm). The animals were anesthetized as previously described. The ICG and Ferric NP containing liposomes were injected via the tail vein. The MESL liposomes without treatment were used as a control. As a comparison, MESL liposomes pretreated with 20 min of SMase enzyme and 5 min of AMF (2 mT 1.6 kA/m 100 kHz) were injected in the tail vein. The leg artery was used as a point of analysis for photoacoustic imaging.

### 4.7. Sphingomyelinase Activity Measurements

SCC tumors were collected, and the ASMase activity was analyzed using an Amplex Red reagent-based fluorometric kit. The assay was performed in acidic conditions. For comparison, the ASMase activity values were analyzed from mouse muscles.

## 5. Conclusions

We tested the tumor site targeting and release concept using sphingomyelin-containing liposomes encapsulated with indocyanine green, fluorescent marker, or the anticancer drug cisplatin. We engineered the liposomes by adding paramagnetic beads to act as a receiver of outside given magnetic energy. Our explanation of the release mechanism relies on our SAXS, TEM, and drug release assay data. We conclude that the ASMase converts the SM-lipid to ceramide formin microdomains on liposomes. These microdomains cause the iron particles to agglomerate. The membrane destabilization of liposomes by ceramide leads to the aggregation of iron nanoparticles. The agglomerated iron nanoparticles on microdomains respond to the low magnetic energy, which leads to further destabilization of the liposome. This causes liposomes to release their contents. The developed multifunctional liposomes were tested in cell internalization studies, showing calcein release from the liposomes. There we showed that ICG and cisplatin are released. Finally, we studied the MESL system with cisplatin drug in a mouse bearing orthotopic human SCC tongue tumor model and showed targeting of the liposomes and increased survival of the mice treated with MESL liposomal nanoparticles and AMF.

## Supplementary Materials

The following are available online at https://www.mdpi.com/2072-6694/12/12/3767/s1, Figure S1. The different power absorption values of MESL liposomes with different 5nm iron magnetic beads concentrations / constant lipid concentrations. The SPA values increase while iron particle lipid ratio goes down. Figure S2. SAXS data SM liposomes with Fe3O4 nanoparticles in Kratky plot (q2I(q) vs q). Kratky/Ruhland graph shows that in solution of 5nm iron nanoparticles significant aggregation can be seen, from the two maxima which corresponds to characteristic distance of 10 nm i.e., diameter of expected nanoparticles which are well packed in larger aggregates. Figure S3. SAXS spectra of SM liposomes with NPs (blue), AMF treatment (red), SMase treatment (green), AMF&SMase treatment (dark blue). Figure S4. SAXS spectra. Blue line - Indocyanine green liposomes with iron nanoparticles, sample was partly dried and we see formation of crystalline phase in large q. Red line—Indocyanine green liposomes with iron nanoparticles with SMase and AMF treatment. Video S1.
