# Supplementary material for: Lipid-Iron Nanoparticle with a Cell Stress Release Mechanism Combined with a Local Alternating Magnetic Field Enables Site-Activated Drug Release
Source: Cancers (Basel). 2020 Dec 14;12(12):3767. doi: 10.3390/cancers12123767 (PMC7765112; doi:10.3390/cancers12123767)
Supplement: Supplementary file 1 [file cancers-12-03767-s001.zip › supplementary materials/cancers-1022541-suppl.pdf]

cancers

Supplementary Materials: Lipid-iron nanoparticle with a cell stress release mechanism combined with a local alternating magnetic field enables site-activated drug release

Tuula Peñate Medina, Mirko Gerle, Jana Humbert, Hanwen Chu, Anna Köpnick, Reinhard Barkmann, Vasil M. Garamus, Bea Sanz, Nikolai Purcz, Olga Will, Lia Appold, Timo Damm, Juho Suojanen, Philipp Arnold, Ralph. Lucius, Regina Willumeit-Römer, Yahya Açil, Joerg Wiltfang, Gerardo. F. Goya, Claus C. Glüer and Oula Peñate Medina

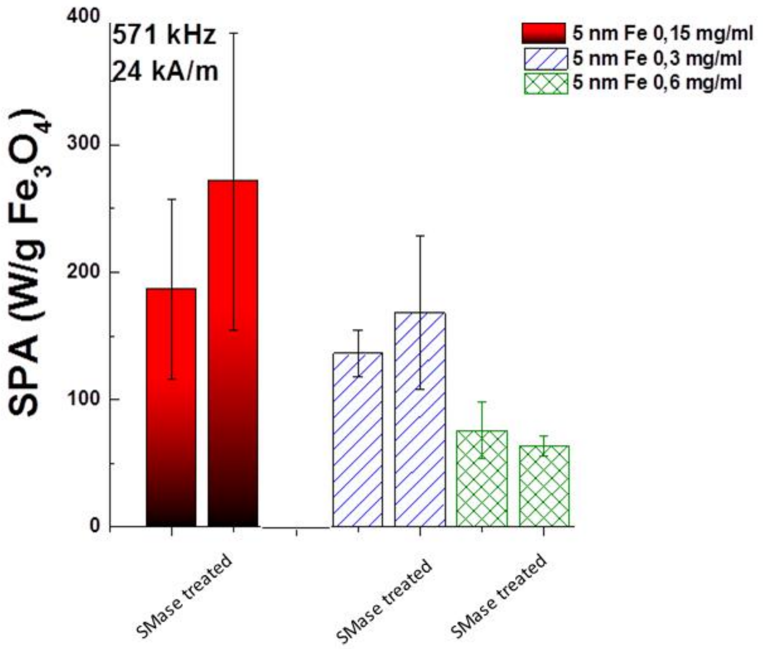

Figure S1. The different power absorption values of MESL liposomes with different 5nm iron magnetic beads concentrations / constant lipid concentrations. The SPA values increase while iron particle lipid ratio goes down.

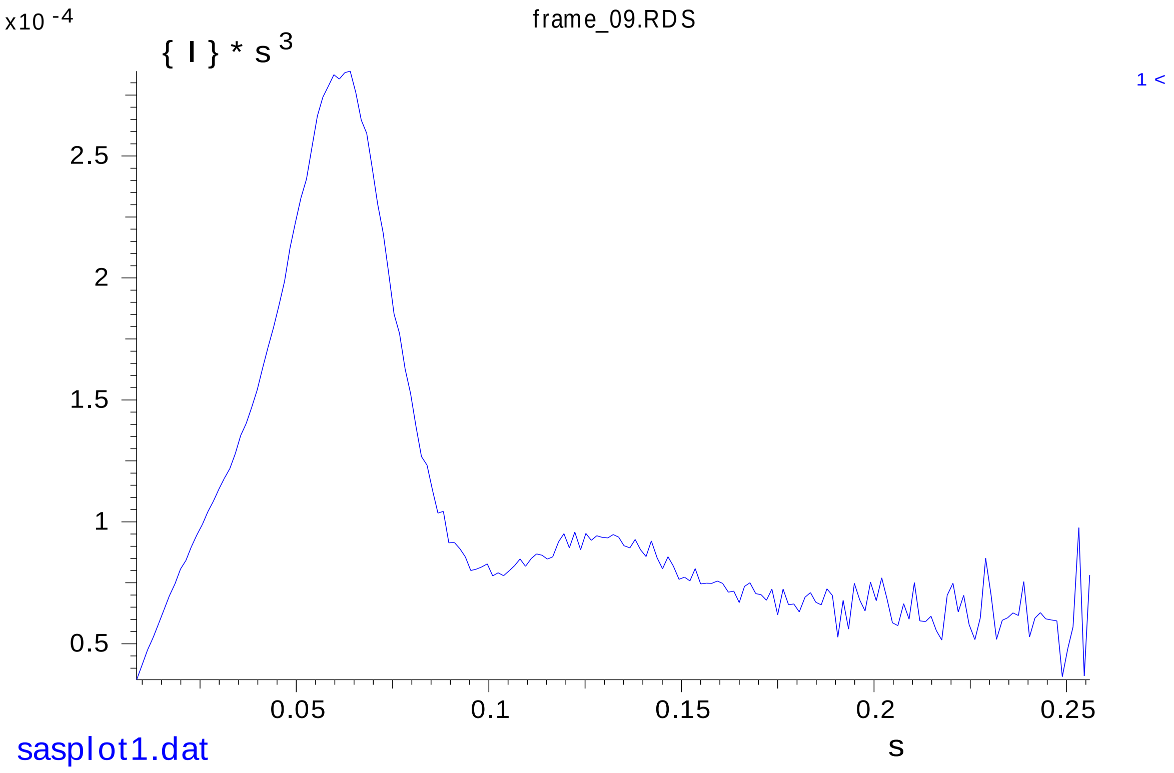

Figure S2. SAXS data SM liposomes with Fe_3O_4 nanoparticles in Kratky plot ($q^2I(q)$ vs q). Kratky/Ruhland graph shows that in solution of 5nm iron nanoparticles significant aggregation can be seen, from the two maxima which corresponds to characteristic distance of 10 nm i.e., diameter of expected nanoparticles which are well packed in larger aggregates.

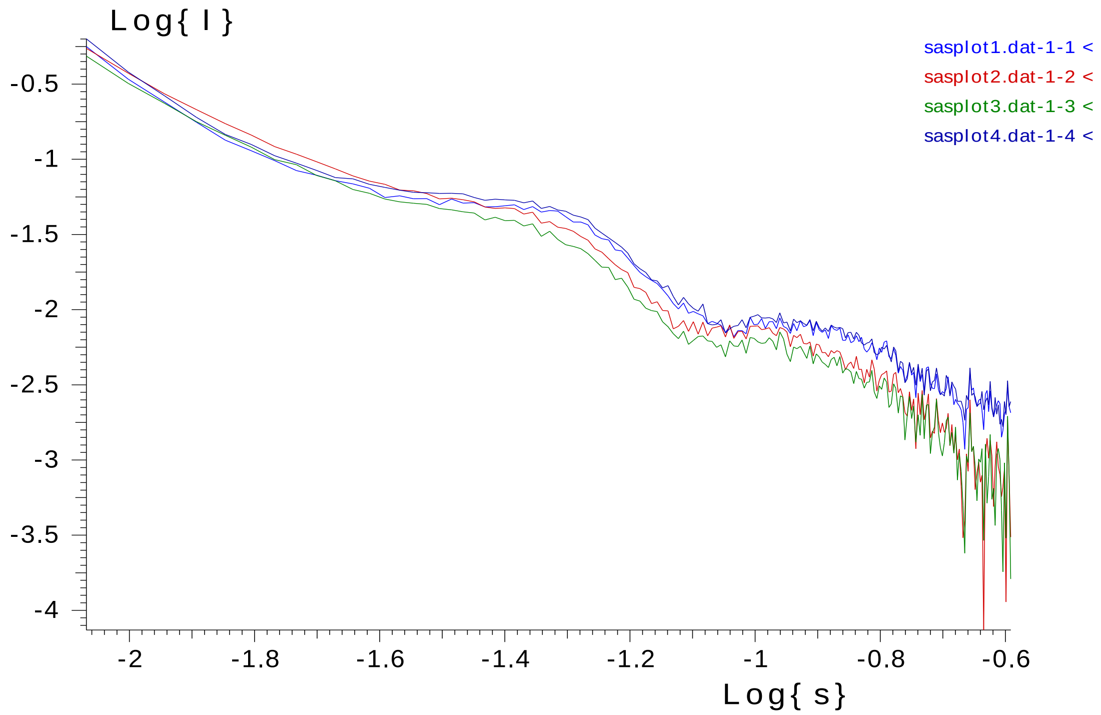

Figure S3. SAXS spectra of SM liposomes with NPs (blue), AMF treatment (red), SMase treatment (green), AMF&SMase treatment (dark blue).

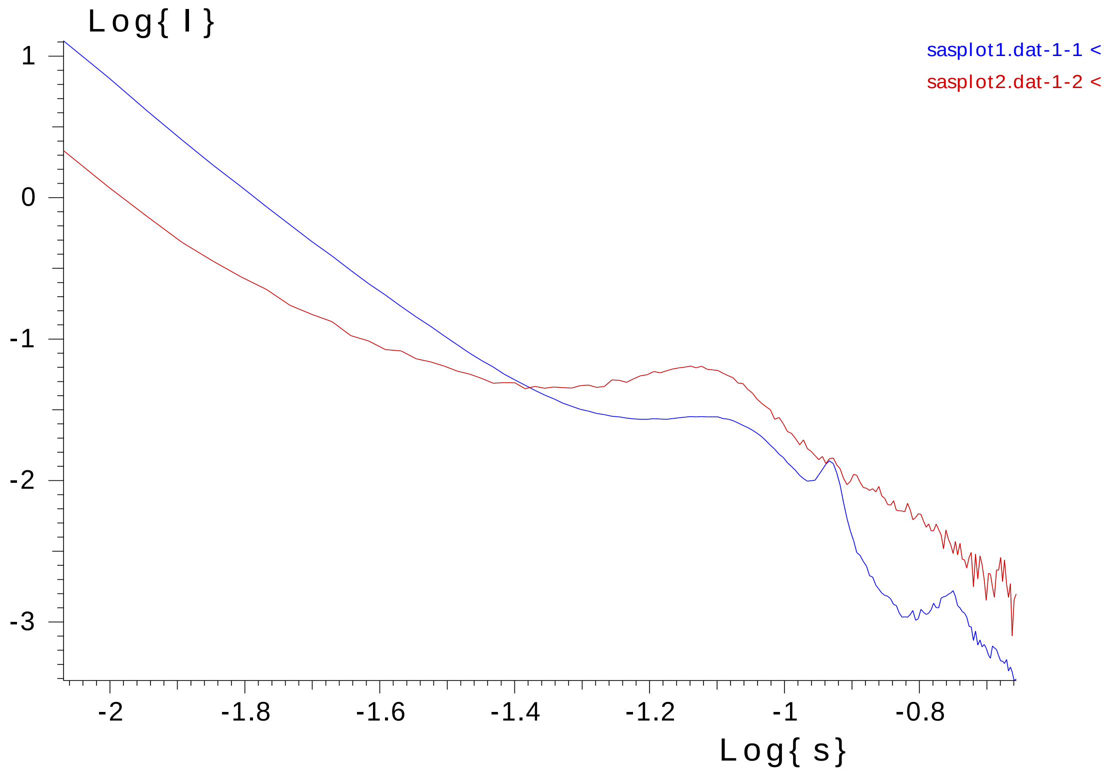

Figure S4. SAXS spectra. Blue line - Indocyanine green liposomes with iron nanoparticles, sample was partly dried and we see formation of crystalline phase in large q. Red line - Indocyanine green liposomes with iron nanoparticles with SMase and AMF treatment.
